# Supplementary material for: Machine Learning Accelerates Crystallization for Structure Determination
Source: Angew Chem Int Ed Engl. 2026 May 4;65(25):e1218503. doi: 10.1002/anie.1218503 (PMC13266958; doi:10.1002/anie.1218503)

checkCIF/PLATON report

Structure factors have been supplied for datablock(s) lcz063

THIS REPORT IS FOR GUIDANCE ONLY. IF USED AS PART OF A REVIEW PROCEDURE FOR PUBLICATION, IT SHOULD NOT REPLACE THE EXPERTISE OF AN EXPERIENCED CRYSTALLOGRAPHIC REFEREE.

No syntax errors found.   CIF dictionary   Interpreting this report

Datablock: lcz063

Bond precision:	C-C = 0.0067 A		Wavelength=1.54184
Cell:	a=17.1674(3)	b=13.1306(2)	c=14.9007(2)
	alpha=90	beta=93.635(1)	gamma=90
Temperature:	100 K		

	Calculated	Reported
Volume	3352.13(9)	3352.13(9)
Space group	I 2/a	I 1 2/a 1
Hall group	-I 2ya	-I 2ya
Moiety formula	C15 H3 Ag3 F18 N6, C14 H8 O3	C15 H3 Ag3 F18 N6, C14 H8 O3
Sum formula	C29 H11 Ag3 F18 N6 O3	C29 H11 Ag3 F18 N6 O3
Mr	1157.05	1157.05
Dx,g cm-3	2.293	2.293
Z	4	4
Mu (mm-1)	15.289	15.289
F000	2216.0	2216.0
F000'	2226.68	
h,k,lmax	21,16,18	20,16,18
Nref	3347	3239
Tmin,Tmax	0.115,0.137	0.562,1.000
Tmin'	0.013	

Correction method= # Reported T Limits: Tmin=0.562 Tmax=1.000
AbsCorr = MULTI-SCAN

Data completeness= 0.968 Theta(max)= 72.714

R(reflections)= 0.0372(3010) wR2(reflections)= 0.1029(3239)

S = 1.063 Npar= 320

The following ALERTS were generated. Each ALERT has the format
test-name_ALERT_alert-type_alert-level.
Click on the hyperlinks for more details of the test.

Alert level C

```
PLAT216_ALERT_3_C Disordered C5#       (An/Solv) ADP max/min Ratio        5.2 Note
PLAT250_ALERT_2_C Large U3/U1 Ratio for <U(i,j)> Tensor(Resd    1)        2.2 Note
PLAT309_ALERT_2_C Single Bonded Oxygen (C-O > 1.3 Ang) ..........          O2 Check
PLAT911_ALERT_3_C Missing FCF Refl Between Thmin & STh/L=    0.600          3 Report
              -13 12  1, -16  0 12,  -2  0 14,
PLAT971_ALERT_2_C Check Calcd Resid. Dens.  1.37Ang From F00D            1.77 eA-3
```

Alert level G

```
PLAT002_ALERT_2_G Number of Distance or Angle Restraints on AtSite          3 Note
PLAT003_ALERT_2_G Number of Uiso or U(i,j)  Restrained non-H-Atoms         17 Report
PLAT007_ALERT_5_G Number of Unrefined Donor-H Atoms .............           1 Report
              H1
PLAT083_ALERT_2_G SHELXL Second Parameter in WGHT  Unusually Large      17.12 Why ?
PLAT172_ALERT_4_G The CIF-Embedded .res File Contains DFIX Records          1 Report
PLAT173_ALERT_4_G The CIF-Embedded .res File Contains DANG Records          1 Report
PLAT174_ALERT_4_G The CIF-Embedded .res File Contains FLAT Records          1 Report
PLAT177_ALERT_4_G The CIF-Embedded .res File Contains DELU Records          1 Report
PLAT178_ALERT_4_G The CIF-Embedded .res File Contains SIMU Records          2 Report
PLAT186_ALERT_4_G The CIF-Embedded .res File Contains ISOR Records          1 Report
PLAT188_ALERT_3_G A Non-default SIMU Restraint Value has been used     0.0100 Report
PLAT188_ALERT_3_G A Non-default SIMU Restraint Value has been used     0.0100 Report
PLAT192_ALERT_3_G A Non-default DELU Restraint Value for SecondPar     0.0200 Report
PLAT242_ALERT_2_G Low    'MainMol' Ueq as Compared to Neighbors of       C00S Check
PLAT242_ALERT_2_G Low    'MainMol' Ueq as Compared to Neighbors of       C00T Check
PLAT242_ALERT_2_G Low    'MainMol' Ueq as Compared to Neighbors of       C00U Check
PLAT299_ALERT_4_G Atom Site Occupancy Constrained at ............         0.5 Check
              O1      O007    O2      C1      C2      C3      C4      C5
              C00P    C6      C7      C00R    C00N    C00I    C00K    C00O
              C00L    H1      H3      H00R    H00O    H5      H00L    H6
              H7
PLAT302_ALERT_4_G Anion/Solvent/Minor-Residue Disorder (Resd    2)       100% Note
PLAT304_ALERT_4_G Non-Integer Number of Atoms in ..... (Resd    2)      12.50 Check
PLAT333_ALERT_2_G Large Aver C6-Ring C-C Dist C1       -C00N     .       1.44 Ang.
PLAT432_ALERT_2_G Short Inter X...Y Contact  F00E     ..C00P     .       2.96 Ang.
                                                     x,y,z  =      1_555 Check
PLAT720_ALERT_4_G Number of Unusual/Non-Standard Labels .........          33 Note
              Ag01    Ag02    F003    F004    F005    F006    O007    F00A
              N00B    F00C    F00D    F00E    C00F    H00F    F00G    C00H
              C00J    C00M    C00P    C00Q    H00Q    C00R    H00R    C00N
              C00I    C00K    C00O    H00O    C00L    H00L    C00S    C00T
              C00U
```

```
PLAT789_ALERT_4_G Atoms with Negative _atom_site_disorder_group  #         25 Check
PLAT811_ALERT_5_G No ADDSYM Analysis: Too Many Excluded Atoms ....          ! Info
PLAT822_ALERT_4_G CIF-embedded .res Contains Negative PART Numbers          4 Check
PLAT860_ALERT_3_G Number of Least-Squares Restraints ............         280 Note
PLAT883_ALERT_1_G Absent Datum for _atom_sites_solution_primary ..     Please Do !
PLAT912_ALERT_4_G Missing # of FCF Reflections Above STh/L=  0.600        102 Note
PLAT941_ALERT_3_G Average HKL Measurement Multiplicity ..........         3.8 Low
PLAT969_ALERT_5_G The 'Henn et al.' R-Factor-gap value ..........       5.599 Note
              Predicted wR2: Based on SigI**2  1.84 or SHELX Weight  9.68
PLAT978_ALERT_2_G Number C-C Bonds with Positive Residual Density.          3 Info
```

```
   0 ALERT level A = Most likely a serious problem - resolve or explain
   0 ALERT level B = A potentially serious problem, consider carefully
   5 ALERT level C = Check. Ensure it is not caused by an omission or oversight
  31 ALERT level G = General information/check it is not something unexpected

   1 ALERT type 1 CIF construction/syntax error, inconsistent or missing data
  12 ALERT type 2 Indicator that the structure model may be wrong or deficient
   7 ALERT type 3 Indicator that the structure quality may be low
  13 ALERT type 4 Improvement, methodology, query or suggestion
   3 ALERT type 5 Informative message, check
```

Validation response form

Please find below a validation response form (VRF) that can be filled in and pasted into your CIF.

```
# start Validation Reply Form
_vrf_PLAT216_lcz063
;
PROBLEM: Disordered C5#       (An/Solv) ADP max/min Ratio        5.2 Note
RESPONSE: ...
;
_vrf_PLAT250_lcz063
;
PROBLEM: Large U3/U1 Ratio for <U(i,j)> Tensor(Resd    1)        2.2 Note
RESPONSE: ...
;
_vrf_PLAT309_lcz063
;
PROBLEM: Single Bonded Oxygen (C-O > 1.3 Ang) ..........          O2 Check
RESPONSE: ...
;
_vrf_PLAT911_lcz063
;
PROBLEM: Missing FCF Refl Between Thmin & STh/L=    0.600          3 Report
RESPONSE: ...
;
_vrf_PLAT971_lcz063
;
PROBLEM: Check Calcd Resid. Dens.  1.37Ang From F00D           1.77 eA-3
RESPONSE: ...
;
# end Validation Reply Form
```

It is advisable to attempt to resolve as many as possible of the alerts in all categories. Often the minor alerts point to easily fixed oversights, errors and omissions in your CIF or refinement strategy, so attention to these fine details can be worthwhile. It is up to the individual to critically assess their own results and, if necessary, seek expert advice.

PLATON version of 17/09/2025; check.def file version of 17/09/2025

---

duplicate check

No duplication found

Datablock lcz063 - ellipsoid plot

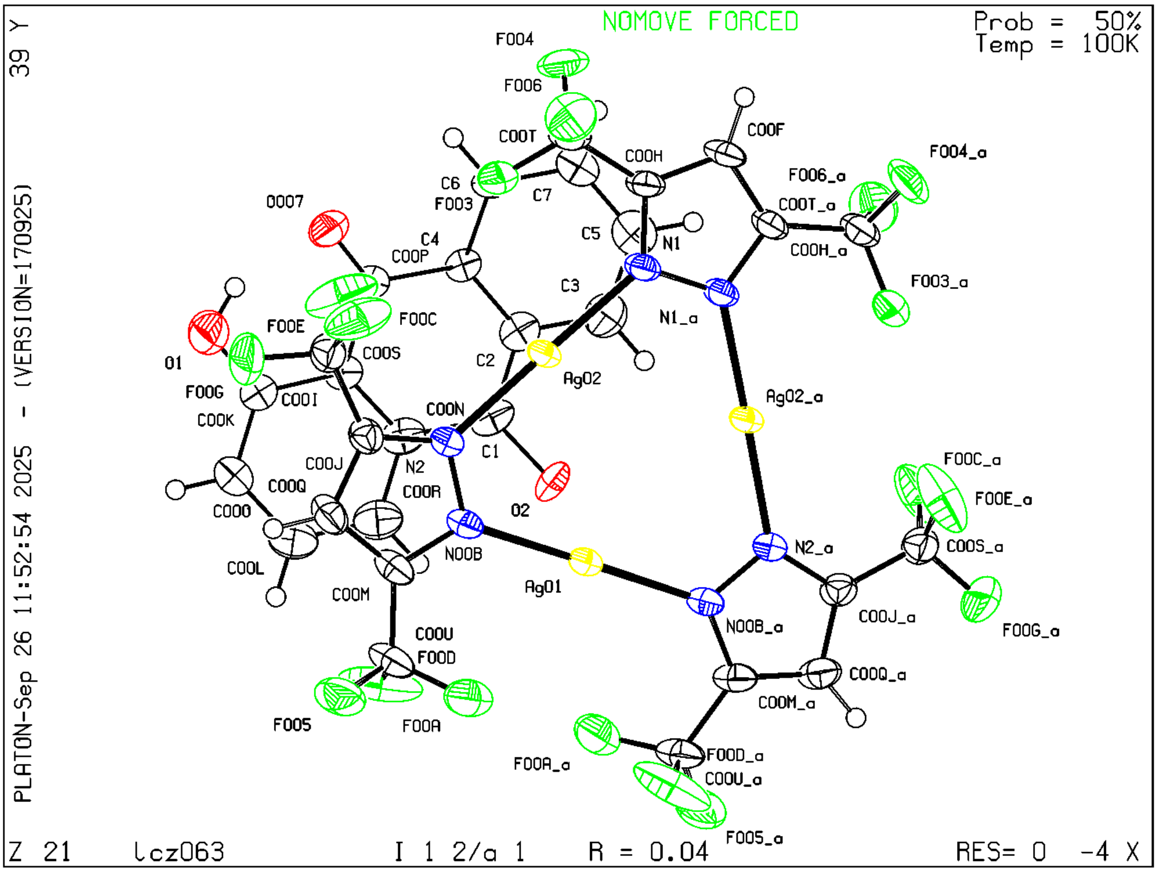

Supplement: Supplementary file 2 — Supporting File 2: anie72462‐sup‐0002‐DataFile.zip. [file ANIE-65-e1218503-s002.zip › supplementary_materials_crystallographic_data submit.zip!/cif_and_checkcif(1-114)/Ag3Pz3_63/Ag3Pz3_63_checkcif.pdf]
